# Thermal stress-induced metabolic reprogramming in two hard coral species

**DOI:** 10.1016/j.isci.2026.116207

**Published:** 2026-06-08

**Authors:** Enrico Montalbetti, Tecla Aramini, Marcella Bonanomi, Yohan Didier Louis, Elisa Brivio, Leilei Zhang, Pascual Garcia Perez, Danilo Porro, Luigi Lucini, Silvia Lavorano, Davide Seveso, Paolo Galli, Daniela Gaglio

**Affiliations:** 1 Department of Earth and Environmental Sciences (DISAT), University of Milano - Bicocca, Piazza della Scienza 1, 20126 Milano, Italy; 2 MaRHE Center (Marine Research and High Education Centre), Magoodhoo Island, Faafu Atoll, Maldives; 3 NBFC (National Biodiversity Future Center), 90133 Palermo, Italy; 4 Institute of Bioimaging and Biological Complex Systems (IBSBC), National Research Council (CNR), Rome, Italy; 5 New York University Abu Dhabi, Saadiyat Island, Abu Dhabi, United Arab Emirates; 6 Mubadala Arabian Center for Climate and Environmental Sciences, Abu Dhabi, United Arab Emirates; 7 Department of Biotechnology and Biosciences (BTBS), University of Milano - Bicocca, Piazza della Scienza, 2, 20126 Milano, Italy; 8 Department for Sustainable Food Process – DiSTAS, Università Cattolica del Sacro Cuore, 29122 Piacenza, Italy; 9 Department of Food Technology, Nutrition, and Food Science, Veterinary Faculty, University of Murcia, Regional Campus of International Excellence “Campus Mare Nostrum”, 30100 Murcia, Spain; 10 Costa Edutainment SpA - Acquario di Genova, Area Porto Antico, Ponte Spinola, 16128 Genoa, Italy; 11 University of Dubai, P. O. Box 14143, Dubai, United Arab Emirates

**Keywords:** Environmental science, Zoology, Metabolomics

## Abstract

Coral reefs are increasingly impacted by marine heatwaves that disrupt the symbiosis between corals and their symbiotic dinoflagellates. Using untargeted LC-MS metabolomics, we investigated heat-stress responses at the coral holobiont level in *Stylophora pistillata* (*S. pistillata*) and *Pocillopora damicornis* (*P. damicornis*) from the northern Red Sea. Under control conditions (25°C), the two species exhibited distinct baseline metabolic profiles, indicating different energy-metabolism strategies. After 10 days at 31°C, both the corals showed pronounced metabolic reprogramming but with contrasting responses, *P. damicornis* increased amino acid metabolism, redox buffering, and ammonia recycling, consistent with enhanced cellular defense. In contrast, *S. pistillata* reduced central carbon metabolism and shifted toward alternative energy pathways and lipid remodeling. These findings show that closely related corals can adopt divergent holobiont-level metabolic strategies under thermal stress, highlighting metabolic plasticity as an important component of coral responses to ocean warming.

## Highlights

• Thermal stress drives species-specific coral metabolic reprogramming
• Closely related coral species showed distinct baseline metabolism
• *Pocillopora damicornis* enhances redox buffering and amino acid metabolism
• *Stylophora pistillata* shifts toward lipid remodeling and alternative pathways

## Graphical abstract

**Figure:**
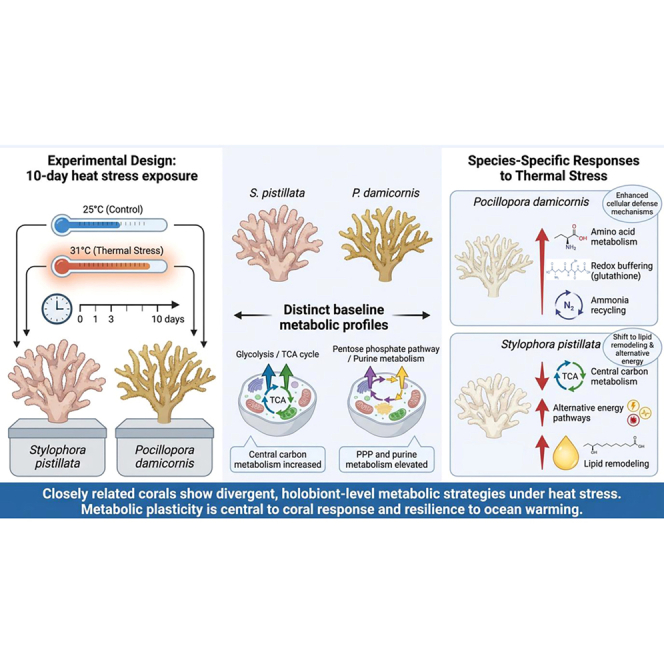

## Introduction

Global degradation of tropical coral reefs has become one of the most concerning phenomena associated with global climate change. Indeed, coral reefs have declined by at least 50% over the past 50 years in large parts of the world’s tropical regions, and most of this decline is connected to global warming-induced coral bleaching events.^1^ Marine heatwaves are the leading cause of coral bleaching, becoming more intense and frequent over the last few decades and causing increases in average water temperatures on a regional and global scale.^2^^,^^3^^,^^4^^,^^5^^,^^6^ Since 1998, four global mass bleaching events have been reported (1998; 2010; 2014–2017; 2023–2024), hitting the majority of the planet’s reefs, causing levels of coral mortality of up to 98% and local extinction of several coral species in different locations.^7^^,^^8^^,^^9^^,^^10^ Coral bleaching is the breakdown of the mutualistic symbiosis between cnidarian hosts and their photosynthetic dinoflagellate endosymbionts (family Symbiodiniaceae), resulting in symbiont loss and exposure of the white color of the underlying calcium carbonate skeleton.^11^^,^^12^ Symbionts provide photosynthetic products to their coral host, covering up to 90% of the host’s energy requirements.^13^ When symbionts are lost from coral host tissues, the coral is deprived of essential photosynthates, which may lead to starvation.^14^ If the mutualism is not restored within weeks to months, the coral will die.^14^^,^^15^

The molecular mechanisms underlying coral bleaching are not yet fully understood, and different hypotheses have been proposed.^15^ In this context, the oxidative hypothesis and the carbon limitation hypothesis are currently the most widely accepted.^15^^,^^16^^,^^17^ The first proposes that the accumulation of reactive oxygen species (ROS) and reactive nitrogen species (RNS), both at the symbiont and host levels under stress conditions, may overwhelm the host’s physiological response mechanisms.^18^^,^^19^^,^^20^ This could lead to non-specific damage to cellular components, such as DNA, proteins, and lipids, and ultimately to the loss of symbionts via processes including host-cell apoptosis and symbiont exophagy/autophagy.^21^^,^^22^^,^^23^ Conversely, the carbon limitation hypothesis posits that increased symbiont photosynthetic activity under high temperatures and irradiance reduces CO_2_ availability in coral cells.^24^ As a result, the photosynthetic apparatus of dinoflagellates undergoes photoinhibition, leading to increased ROS and RNS and, consequently, the expulsion of symbionts.^25^^,^^26^ These processes may have detrimental effects on the metabolic performance of the coral holobiont since the remaining symbiont cells could benefit from increased nitrogen availability from the host, causing the retention of more photosynthates to be exploited for growth, thereby potentially limiting the energetic resources available to the host during bleaching events.^17^^,^^26^^,^^27^^,^^28^ As an integrative explanation, coral bleaching may be understood as a breakdown in metabolic compatibility between symbionts and hosts.^8^ However, the mechanisms underlying this metabolic incompatibility remain poorly investigated, and much remains to be clarified regarding the metabolic processes involved in coral symbiosis.^15^

Corals show different responses to heat stress and variable bleaching patterns over space and time depending on various factors, such as the duration and frequency of thermal anomalies, site-specific environmental conditions, and their ecological and thermal history.^29^^,^^30^^,^^31^^,^^32^^,^^33^ However, intrinsic factors such as morphological, molecular, and physiological aspects of the coral holobiont seem to play a key role in determining the different thermotolerance among and within species.^34^^,^^35^^,^^36^^,^^37^ In particular, the physical characteristics of coral tissues and skeletal structures,^38^^,^^39^^,^^40^^,^^41^ the host and symbiont genotype,^6^^,^^42^^,^^43^^,^^44^^,^^45^ the microbiome composition^46^^,^^47^^,^^48^ and the defense mechanisms at the cellular and molecular level involving gene expression and proteins^49^^,^^50^^,^^51^^,^^52^^,^^53^ are the main and most investigated hallmarks of the variable responses to bleaching.

In recent years, a new “omics” technique, metabolomics, has demonstrated its potential for investigating metabolic processes and constitutes an innovative tool with numerous applications across fields.^54^^,^^55^ Metabolomics confers a high-throughput qualitative and quantitative perspective of all metabolites in biological samples, such as cells, tissues, biofluids, and organisms.^56^ The complete set of metabolites and their interactions in a determinate biological system is known as the metabolome, which depends on the genome and other factors such as environmental conditions, diseases, or exposure to stressors.^55^ To date, metabolomics in coral studies has been mainly adopted to investigate coral disease impact/mechanisms, to understand the host-symbiont relationship better, or to identify new biomarkers to be used in physiological studies.^57^^,^^58^^,^^59^^,^^60^ However, compared to other “omics,” metabolomic research on corals is only in its infancy, and few studies have focused on the prolonged effects of heat stress on the metabolome of different species.

Recent metabolomic studies have begun to elucidate how coral holobionts reorganize their metabolic networks in response to environmental stressors, including thermal stress, habitat variability, and disease. Untargeted metabolomics analyses have reported increases in free amino acids and small peptides in thermally stressed or bleaching corals, consistent with altered protein turnover and stress-response processes, in genera such as *Acropora*, *Pocillopora*, and *Montipora*.^61^^,^^62^ Enhanced abundances of redox-related metabolites, including glutathione and associated intermediates, have been observed in corals thriving in extreme or highly variable thermal environments, suggesting an increased capacity for redox buffering under chronic stress exposure.^61^ Lipid remodeling, including shifts in fatty acid composition and accumulation of specific mono- and poly-unsaturated fatty acids, has also been documented under thermal stress and across contrasting reef habitats, potentially reflecting changes in membrane properties and energy storage strategies.^63^^,^^64^ In addition, a diverse array of specialized metabolites, such as flavonoids, phenylpropanoids, and other secondary compounds, has been detected in coral holobionts and is thought to originate largely from symbiotic dinoflagellates or associated microbial communities, particularly under stress conditions.^63^ Together, these studies demonstrate the power of metabolomics to capture integrative biochemical responses of coral holobionts to environmental stress, underscoring a largely exploratory nature of this technique.

In this study, we adopted a metabolomics approach to investigate the response of two of the main widespread coral species in Indo-Pacific reefs, *Pocillopora damicornis* (*P. damicornis*) and *Stylophora pistillata* (*S. pistillata*) originating from the northern Red Sea, to a 10-day of controlled laboratory thermal stress, reproducing a standard stress experimental profile, rather than replicating a specific *in situ* marine heatwave event. We hypothesized that the two corals would exhibit bleaching during the experimental stress period, as reflected in a temporal metabolic signature of the holobiont. To test this hypothesis, our first goal was to characterize the bleaching levels of the two species under stress by analyzing symbiont density and chlorophyll content at different time points. We then aimed to compare metabolomic differences between the two species and across time points within the same species. We specifically examined metabolic and stress-response pathways that corals might upregulate or downregulate in relation to bleaching severity. Expanding our knowledge of the metabolic pathways involved in bleaching responses and understanding which strategies different species adopt in response to stress at the metabolic level can further clarify differences in bleaching susceptibility among corals and potentially assist in selecting resistant ones.

## Results

### Coral bleaching assessment

#### Chl *a* concentration

**Figure 1:**
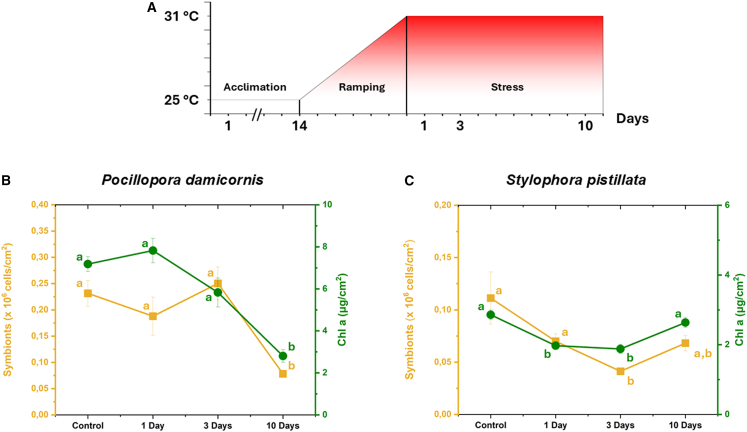
Experimental temperature profile and Symbiodiniaceae and chlorophyll content (A) Schematic representation of the different experimental phases with related temperature indication and exposure days. During the thermal stress, corals were sampled at 0 (control), 1, 3, and 10 days. (B and C) Levels of symbiont density and chlorophyll *a* concentration at different time points in *P. damicornis* (B) and *S. pistillata* (C). Significant statistical differences are indicated by letters (a; b). Data at each time point are presented as mean ± SEM.

Chlorophyll *a* (Chl *a*) concentration showed different patterns in the two investigated species. In *P. damicornis*, no significant change in Chl *a* level was observed in the first 3 days of stress (Figure 1B), while a significant decrease in Chl *a* concentration was detected at 10 days. In *S. pistillata*, the Chl *a* concentration significantly decreased compared to the control already after 1 day of stress, remaining constant without further significant change until 3 days into the experiment (Figure 1C). Unexpectedly, Chl *a* concentration returned to values significantly higher than those recorded at 1 and 3 days and similar to those of the control group after 10 days of stress. No statistically significant changes in Chl *a* concentration were observed over time in control corals maintained at 25°C throughout the experimental period (Figure S2).

#### Symbiodiniaceae density

Symbiodiniaceae density showed different trends in the two investigated species. In *P. damicornis*, symbiont density fluctuated during the first 3 days of stress, without any significant change relative to the control (Figure 1B). In particular, density decreased after 1 day of stress and returned to levels similar to the control after 3 days. After 10 days of stress, Symbiodiniaceae density was significantly lower than at previous time points and the control, indicating a consistent loss of zooxanthellae from coral tissues. On the other hand, *S. pistillata* experienced a quick decrease in Symbiodiniaceae density, reaching a lower level after 3 days (Figure 1C). Indeed, after 10 days, Symbiodiniaceae density slightly increased, showing a rise in symbiont density in coral tissues.

No statistically significant changes in Symbiodiniaceae density were observed over time in control corals maintained at 25°C across the experimental duration (Figure S2).

### Symbiodiniaceae identity

All the *P. damicornis* mother colonies exclusively hosted dominant Symbiodiniaceae belonging to the genus *Cladocopium* (previously clade C; NCBI accession numbers PP264470-PP264477), whereas all the *S. pistillata* mother colonies hosted dominant Symbiodiniaceae of the genus *Symbiodinium* (previously clade A; NCBI accession numbers PP264478-PP264485). In both cases, no within-species genetic diversity was detected.

### Metabolic profiling

#### Metabolic profiling in control conditions

All metabolomics raw data are available in the following repository: https://osf.io/t4xd9/?view_only=4276e8e53c294a5fa4165eae888ed2eb.

**Figure 2:**
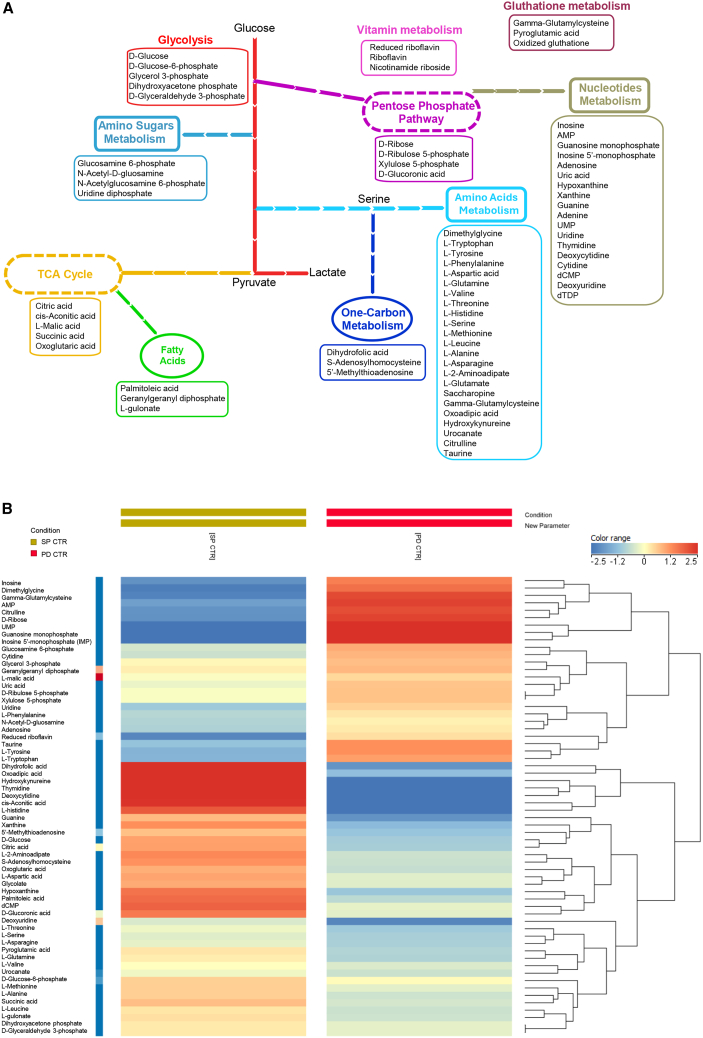
Metabolic profiling of *P. damicornis* and *S. pistillata* in control condition (A) Schematic representation of primary metabolic pathways and their relative metabolites present in the text. (B) Hierarchical clustering heatmaps showing significantly different intracellular metabolites in *P. damicornis* control vs. *S. pistillata* control, as detected by LC-MS. Colors represent different levels that increase from blue to red.

Metabolomic profiles obtained after 1, 3, and 10 days of thermal exposure were compared to the pre-stress baseline (time 0) to quantify stress-induced metabolic reprogramming within each species. Metabolic profiling of *P. damicornis* and *S. pistillata* indicated that the two species were phenotypically distinct. A schematic representation of the metabolites and their relative pathways analyzed in this study is shown in Figure 2A. Preliminary analyses of control samples revealed that each species showed a distinct metabolic profile, demonstrating a species-specific set of metabolic pathways (Figure S3). The detailed analysis of the basal metabolism of the control condition highlighted a phenotypical contrast between the two species since glycolysis, tricarboxylic acid (TCA) cycle, and pyrimidine metabolism were upregulated in *S. pistillata* compared to *P. damicornis*, while metabolites implicated in the pentose phosphate pathway (PPP) and purine metabolism were higher in *P. damicornis* (Figure 2B).

#### Metabolic profiling in stress conditions

**Figure 3:**
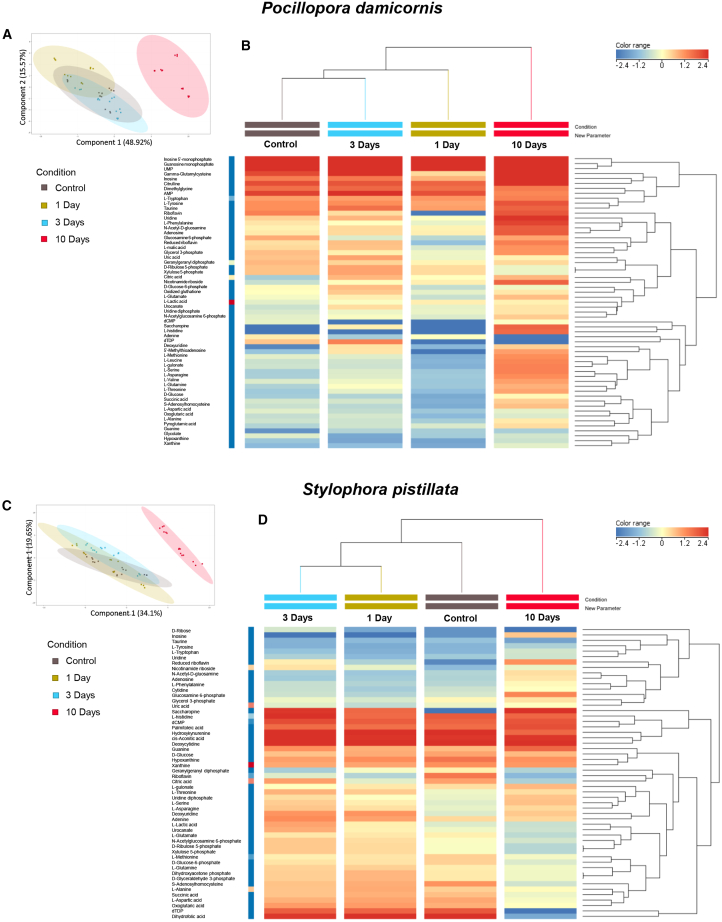
Metabolic characterization of *P. damicornis* and *S. pistillata* at different times of thermal stress (A) Principal component analysis (PCA) plot of *P. damicornis* samples in control and in stress (1, 3, and 10 days) conditions. Gray, control; yellow, 1-day exposure; light blue, 3 days exposure; red, 10 days exposure. (B) Hierarchical clustering heatmaps showing significantly different intracellular metabolites in *P. damicornis* in various conditions (control, 1, 3, and 10 days), as detected by LC-MS. Colors represent different levels that increase from blue to red. (C) Principal component analysis (PCA) plot of *S. pistillata* samples in control and in stress (1, 3, and 10 days) conditions. Gray, control; yellow, 1-day exposure; light blue, 3 days exposure; red, 10 days exposure. (D) Hierarchical clustering heatmaps showing significantly different intracellular metabolites in *S. pistillata* in various conditions (control, 1, 3, and 10 days), as detected by LC-MS. Colors represent different levels that increase from blue to red.

**Figure 4:**
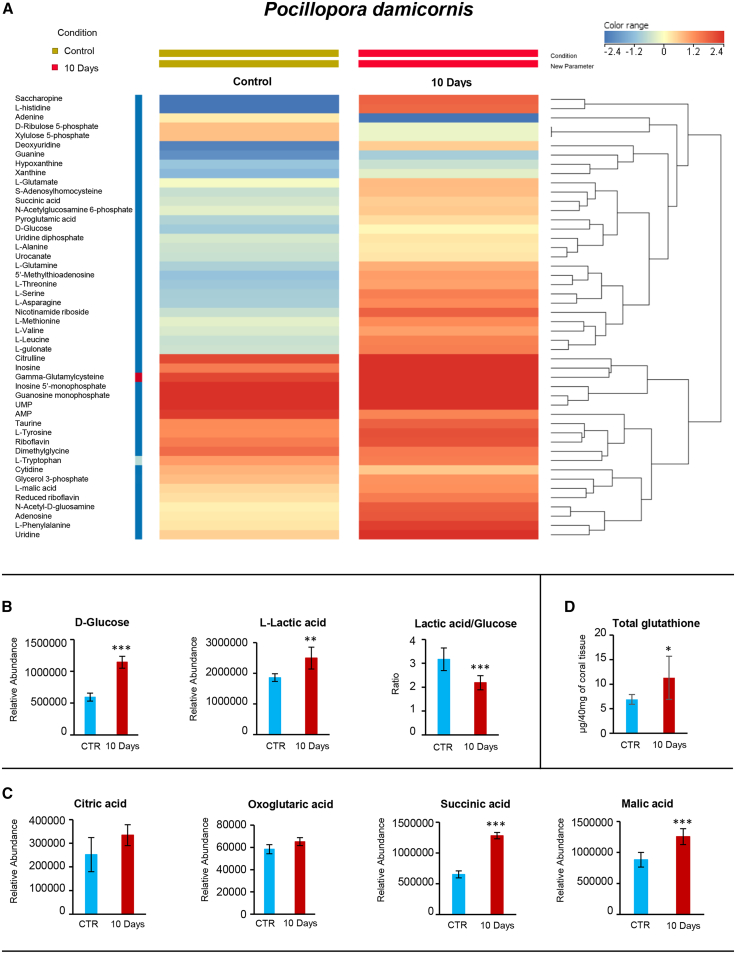
Metabolic profiling comparison between the control condition and the 10-day exposure to the stress condition in *P. damicornis* (A) Hierarchical clustering heatmaps showing significantly different intracellular metabolites in *P. damicornis* control vs. *P. damicornis* 10-day sample as detected by LC-MS. Colors represent increasing levels, progressing from blue to red. (B) Glucose, lactic acid abundance, and lactic acid/glucose ratio in *P. damicornis* control and *P. damicornis* 10-day sample based on relative abundance obtained by LC-MS analysis. (C) Relative metabolite abundance of TCA cycle pathways in *P. damicornis* control and after 10 days of exposure to stress conditions, obtained by LC-MS analysis. (D) Total glutathione measurement in *P. damicornis* control and *P. damicornis* 10 days obtained using Glutathione Assay Kit. Data in (B)–(D) are presented as means ± SD. ∗*p* ≤ 0.1, ∗∗*p* ≤ 0.05, ∗∗∗*p* ≤ 0.01; (*n* = 15).

A distinctive metabolic signature was observed following the longest thermal exposition of 10 days in both species (Figure 3). Indeed, principal-component analysis (PCA) revealed that metabolomic profiles of corals exposed to 1 and 3 days of elevated temperature clustered closely with control samples, whereas samples exposed to 10 days of thermal stress formed a distinct cluster, indicating a pronounced metabolic shift only after prolonged exposure (Figures 3A and 3C). In *P. damicornis*, as expected, 10 days of stress exposure produced a metabolic signature distinct from that of 1- and 3-day thermal exposures and the control condition. The metabolites that resulted significantly different after 10 days of stress were involved in the TCA cycle, amino acids, redox metabolism, nucleotide, and hexosamine biosynthetic pathways (Figure 3B). In contrast to *P. damicornis*, the hierarchical clustering of *S. pistillata* showed significant metabolites involved in PPP, TCA cycle, redox metabolism, mevalonic, amino acids, fatty acids, and hexosamine biosynthetic pathways (Figure 3D). These results revealed that both species presented a slighter metabolic rewiring compared to the control after 1- and 3-day of stress than the one subject to the longest exposition. However, the metabolic adaptation to longer-duration thermal stress (10 days) differed between the two species. Metabolic profiling of *P. damicornis* after 10 days of stress and in control conditions showed that thermal stress increased metabolites involved in ammonia recycling, one-carbon metabolism, and amino acid metabolism. At the same time, there was a reduction in nucleotide metabolism (Figure 4A). Focusing on anaerobic glucose oxidation, we noted significantly enhanced levels of lactic acid and glucose relative abundance in the 10-day sample compared to the control and a decreased lactic acid/glucose ratio (Figure 4B). Moreover, the metabolites involved in the TCA cycle, such as succinic acid and malic acid, were significantly higher in *P. damicornis* after 10 days of stress compared to the control (Figure 4C). In addition, increased levels of total glutathione were observed in *P. damicornis* after 10 days of stress, compared to the control (Figure 4D).

**Figure 5:**
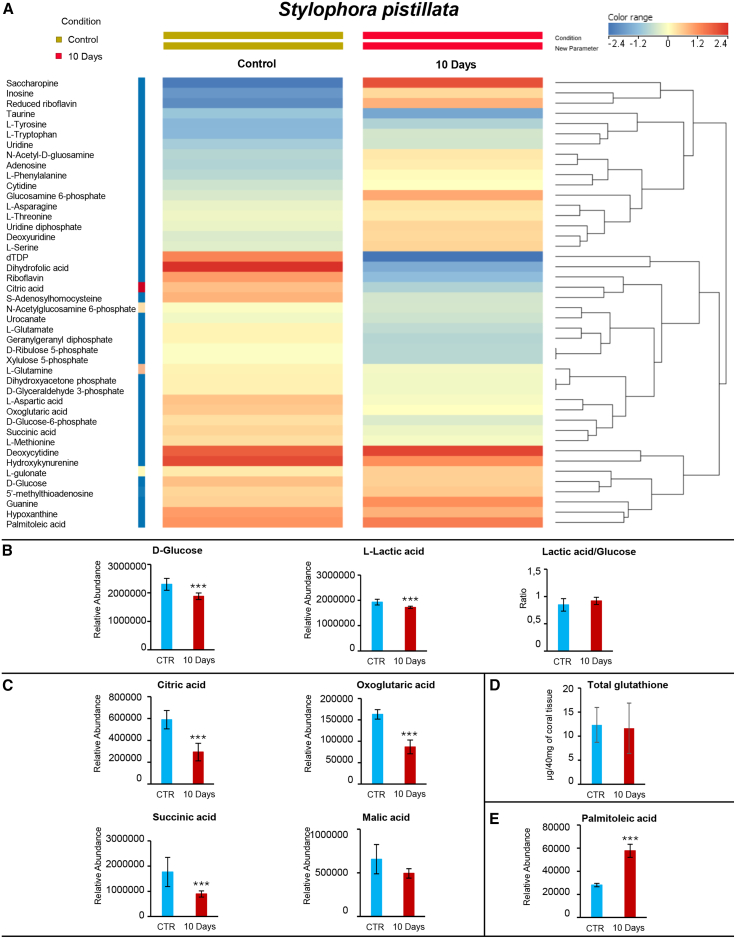
Metabolic profiling comparison between the control condition and the 10 days exposure to stress condition in *S. pistillata* (A) Hierarchical clustering heatmaps showing significantly different intracellular metabolites in *S. pistillata* control vs. *S. pistillata* 10-day sample as detected by LC-MS. Colors represent increasing levels, progressing from blue to red. (B) Glucose, lactic acid abundance, and lactic acid/glucose ratio in *S. pistillata* control and *S. pistillata* 10 days sample based on relative abundance obtained by LC-MS analysis. (C) Relative metabolites abundance of TCA cycle pathways in *S. pistillata* control and after 10 days of exposure to stress conditions, obtained by LC-MS analysis. (D) Total glutathione measurement in *S. pistillata* control and *S. pistillata* 10 days obtained using Glutathione Assay Kit. (E) Relative palmitoleic acid abundance in *S. pistillata* control and after 10 days of exposure to stress conditions, obtained by LC-MS analysis. Data in (B)–(E) are expressed as means ± SD. ∗*p* ≤ 0.1, ∗∗*p* ≤ 0.05, ∗∗∗*p* ≤ 0.01; (*n* = 15).

On the other hand, in *S. pistillata*, an upregulation of pyrimidine metabolism and a down-regulation of PPP, glycolysis, and TCA cycle were noticed (Figure 5A). Regarding the lactic acid, glucose, and lactic acid/glucose ratio, we observed a reduction of glucose and lactic acid abundance in the 10-day sample compared to the control, although no significant difference in the lactic acid/glucose ratio has been found (Figure 5B). Furthermore, the metabolites citric acid, oxoglutaric acid, and succinic acid involved in the TCA cycle were significantly decreased after 10 days of stress compared to the control (Figure 5C). No significant differences have been shown by the analysis of glutathione total levels in *S. pistillata* between the control and 10 days of exposure (Figure 5D). Interestingly, the relative abundance of palmitoleic acid increased after 10 days of thermal stress compared to control conditions.

#### Specialized metabolic profiling of *P. damicornis* and *S. pistillata*

Specialized metabolic profiling was performed using an untargeted approach, confirming that the two coral species exhibited species-specific responses to thermal stress. The comprehensive profiling of the detected features was summarized using a hierarchical cluster analysis (HCA), as shown in Figure S4A. The HCA highlighted a species-specific profile, determined by the two independent clusters, followed by the thermal treatment effect. In detail, the early time points (1 day and 3 days) showed less clear behavior than the 10-day exposure time. The outcome profile confirmed the net metabolic change after long-term exposure instead of the short-term one. The 583 annotated features (Table S2) were then subjected to an enrichment analysis, which revealed the 25 top-enriched classes detected in the coral samples (Figure S4B). Both primary and specialized metabolites characterized corals' overall metabolic classes under thermal stress. Interestingly, carboxylic acids and derivatives, fatty acyls, pyrimidines, and purines were the primary metabolites that were significantly changed, confirming the results mentioned above. Considering the specialized metabolites, polyphenols (flavonoids, coumarins, and tannins), steroids, and isoprenoids were modulated under thermal stress.

As previously determined for the primary metabolites, we conducted separate multivariate analyses for the two coral species to identify species-specific metabolites regulated by the thermal stress response. The first step in the data reduction process for selecting features associated with thermal stress was the use of Boruta, a random forest-based feature selection method. This analysis was conducted for both species, selecting 209 and 155 confirmed features for *P. damicornis* and *S. pistillata*, respectively (Figure S5).

Afterward, processed datasets were subjected to separate multivariate analyses for the two coral species. Regarding *P. damicornis*, the unsupervised HCA discriminated between the starting time points (1 and 3 days) and the prolonged thermal exposure (10 days), placing them in separate clusters from the control samples (Figure S6A). The output was further confirmed by PCA analysis (Figure S6B), which showed that the first two components accounted for nearly 60% of the variance. The same trend was observed for *S. pistillata* coral for both HCA and PCA (Figures S6C and S6D).

**Figure 6:**
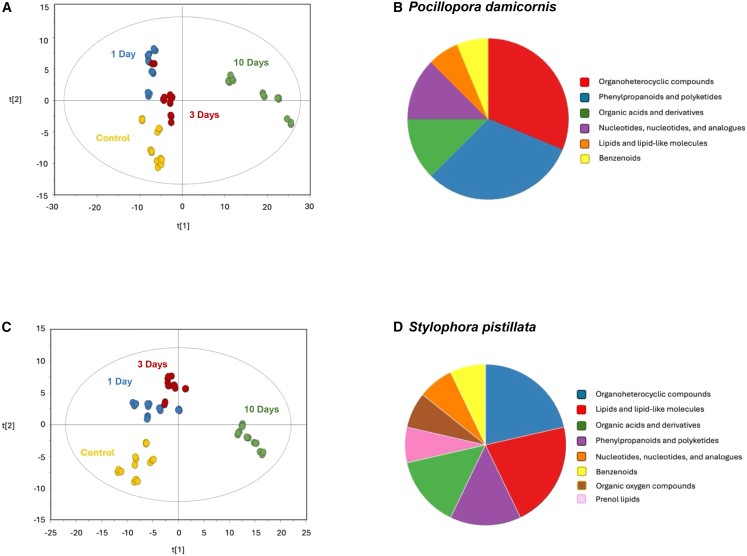
Supervised partial least squares discriminant analysis PLS-DA; (A and C) and variable importance in projection (VIP) (B and D) makers associated with thermal treatments for *P. damicornis* and *S. pistillata* corals

Furthermore, supervised partial least squares discriminant analysis (PLS-DA) was employed in this study to corroborate the unsupervised results and to identify the most discriminative variable importance in projection (VIP) markers associated with thermal treatments (Table S3). The PLS-DA model for *P. damicornis* coral is shown in Figure 6A and was characterized by 96.9% goodness of fit and 94.8% goodness of prediction, and it was cross-validated using ANOVA with *p* values <0.001. The model reported clear discrimination for 10 days of thermal exposure relative to other groups, as indicated by the first vector *t* [1]. The most discriminant VIP markers (VIP scores >1) were selected as important variables and classified based on their compound classes (Figure 6B). Interestingly, the most important variables associated with 10 days of thermal exposure were mainly below the classes of organo heterocyclic compounds, phenylpropanoids, and polyketides, followed by organic acids and derivatives, nucleotides, and lipids. The same trend was observed for *S. pistillata* coral, where the PLS-DA model was characterized by 97.3% goodness of fit and 94.5% goodness of prediction parameters and cross-validated with ANOVA with <0.001 *p* values (Figure 6C). Similarly, the main classes of VIP markers associated with *S. pistillata* coral after 10 days of thermal exposure were organo heterocyclic compounds, organic acids and derivatives, phenylpropanoids, and polyketides (Figure 6D). However, in this last coral species, lipids and lipid-like molecules took a bigger representation compared to *P. damicornis*, including avocadyne, cortisol, and dethiobiotin. Furthermore, organic oxygen compounds and prenol lipids were also reported.

**Figure 7:**
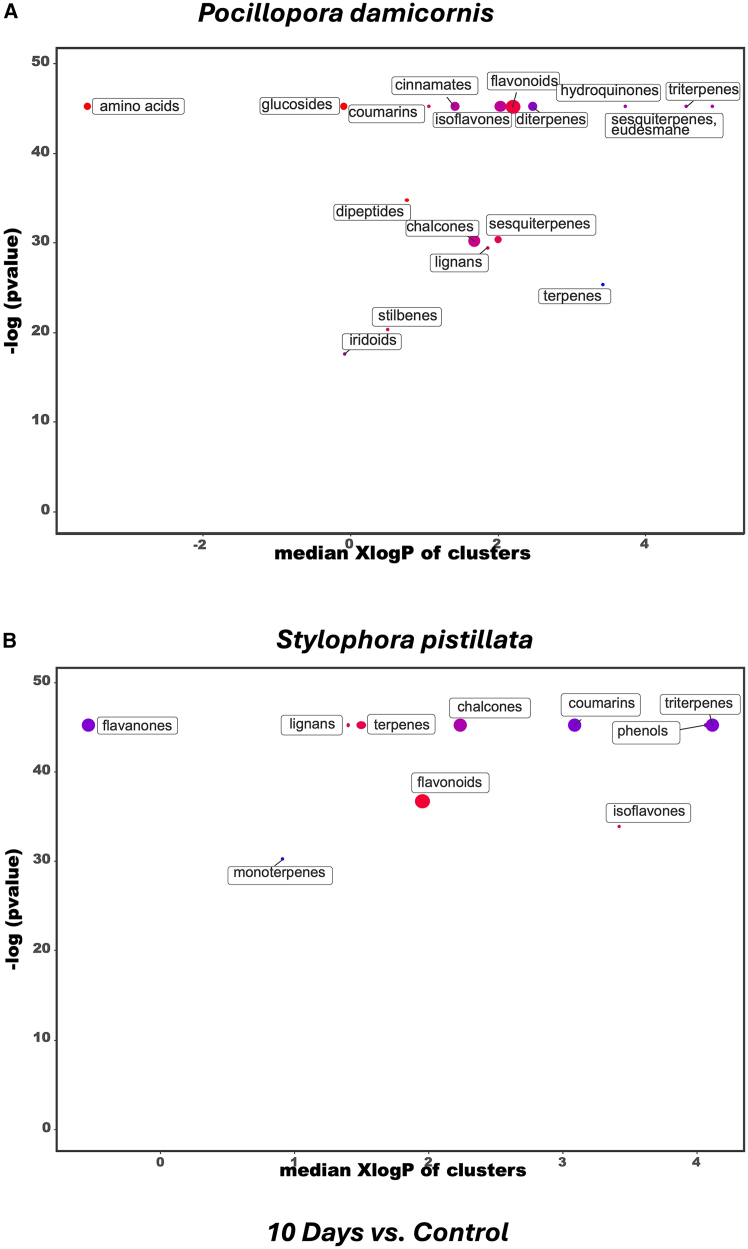
Chemical enrichment analysis of 10 days of thermal exposure compared with the control untreated for *P. damicornis* and *S. pistillata* corals

Given the strong statistical evidence supporting the importance of the 10-day thermal-exposure time point, we focused on this specific metabolic alteration relative to the untreated control. In this sense, volcano analysis (*p* value <0.05 + Fold Change = 2) was used to select the statistically significant and highly changed metabolites deriving from the pairwise comparison between 10 days of thermal exposure vs. control, which were considered for chemical enrichment analysis (ChemRICH) for both *P. damicornis* (Figure 7A) and *S. pistillata* (Figure 7B) corals. Considering *P. damicornis*, the 10 days of thermal exposure determined the increase of protein-degradation compounds, including dipeptides and free amino acids and derivatives (Figure 7A). Moreover, specialized metabolites were observed to be upregulated after thermal exposure, such as flavonoids, coumarins, lignans, and stilbenes, probably due to the presence of symbiotic microorganisms. Specifically, the key compounds per class were phomalone, umbelliferone, epiyangambin, and isorhapontin, respectively. Conversely, terpenoid classes were reported to be down-modulated by the effect of temperature, including di-, tri-, and sesquiterpenes (Table S4). Similar behavior has also been observed in *S. pistillata* (Figure 7B), where flavonoids, lignans, and isoflavones were reported to accumulate at higher levels in thermally treated coral than in the control. On the other hand, monoterpenes and triterpenes were reported to have down-accumulated.

## Discussion

Tropical stony corals are among the most threatened animals on our planet, and their populations, which form unique coral reef ecosystems, have declined 30%–50% compared to pre-industrial times.^65^ The rate of decline of tropical coral reefs has become astonishing since the 1980s, when the first regional-scale coral bleaching events were recorded.^2^^,^^5^ Since then, bleaching events have become increasingly frequent and intense in concomitance with global climate change, and their effects have been exacerbated by many other stressors that led to a general decline of coral reefs.^8^^,^^66^ Despite heat stress-induced coral bleaching being the primary cause of this decline, its mechanisms and the reasons behind different susceptibilities across different species remain unclear, making it difficult to develop proper conservation and mitigation strategies.^15^

In this study, a metabolomics approach was adopted to investigate the responses of two widespread scleractinian coral species, *P. damicornis* and *S. pistillata*, to 10-day heat stress, focusing on the distinct metabolic rearrangements the two species may adopt to cope with increasing ocean warming. In this context, we used untargeted metabolomics on whole-holobiont samples; accordingly, metabolite identities are putative, and the patterns reported here are descriptive associations intended to generate hypotheses for future validation. First, our results showed that the two species exhibited distinct patterns of symbiont loss and a decrease in Chl *a* concentration when subjected to a severe temperature increase, as shown in Figure 1. Corals from the Gulf of Aqaba and northern Red Sea, from where the corals in this study originated, are widely recognized for their comparatively high thermal tolerance, with several studies reporting limited bleaching and modest reductions in symbiont density and Chl *a* content at temperatures that induce bleaching in other reef regions.^67^ Experimental and field-based studies have shown that increases in temperature often lead to gradual declines in chlorophyll concentration prior to substantial symbiont loss, and that prolonged exposure rather than short-term thermal peaks is a key determinant of bleaching severity in these populations.^68^^,^^69^^,^^67^ Nevertheless, in this study, prolonged exposure (10 days) to 31°C under controlled aquarium conditions was sufficient to induce significant bleaching responses, indicating that sustained sub-lethal thermal stress can elicit physiological and metabolic disruption even in corals originating from thermally resilient regions. In this context, *P. damicornis* showed a non-significant fluctuation of Chl *a* and Symbiodiniaceae density in the first 3 days of thermal stress, followed by a marked decrease after 10 days, as shown in Figure 1. Conversely, *S. pistillata* showed a rapid and significant decline in Symbiodiniaceae density and Chl *a* concentration within 1 day of thermal stress, followed by an unexpected recovery of both parameters after 10 days. It is important to specify that the two investigated species hosted in their tissues two different Symbiodiniaceae genera, such as *Cladocopium* (previously clade C) in *P. damicornis* and *Symbiodinium* (previously clade A) in *S. pistillata*, which are known to have different thermotolerance and therefore could have affected the coral bleaching response.^40^^,^^70^ Indeed, different biochemical and physiological aspects of the dinoflagellate cell, such as thylakoid membrane stability in response to heat stress, photosynthetic efficiency, stability of photosystem II, and hydrogen peroxide production,^71^^,^^72^^,^^73^ can modulate the thermal tolerance of different Symbiodiniaceae taxa and potentially influence the holobiont’s ability to cope with stress.^74^ However, several factors beyond symbiont genotypes that affect both the dinoflagellate and the animal, as well as other components of the symbiosis, such as the microbiome, can influence the bleaching susceptibility of hard corals, suggesting that bleaching tolerance is an individual-specific rather than a species-specific process.^34^^,^^75^^,^^76^^,^^77^^,^^78^^,^^79^ In this context, a previous study demonstrated that conspecific corals living in different reef habitats and hosting different Symbiodiniaceae taxa exhibited opposing metabolic responses to heat stress.^80^ In thermally sensitive corals, a greater variation in the metabolic profile of the endosymbionts compared to that of the host was observed. In contrast, heat-resistant corals showed greater metabolic changes in the host than in the symbionts, suggesting that the metabolic relationship between host and endosymbionts may vary even within conspecific individuals and may influence thermal resistance.^80^ Our analysis did not allow us to distinguish between the metabolic profiles of endosymbionts and the host, which may be beneficial for studying responses of various coral populations across different environmental conditions. The coral holobiont comprises a complex consortium of partners, including the cnidarian host, dinoflagellate endosymbionts, and the associated microbiome, all of which can influence heat-stress responses at both the individual and ecosystem levels.^48^^,^^81^ For example, previous evidence indicates that heat stress can alter sugar levels, such as maltose, in the host.^82^ Since maltose is a disaccharide commonly associated with microbial components of the holobiome,^82^ bacteria may play an important role in shaping the host’s metabolome. Therefore, focusing solely on the host and Symbiodiniaceae components, without considering other partners such as the microbiome, may not provide a complete representation of a holobiont’s response to heat stress. For these reasons, in this study, the metabolomic profile of the whole holobiont was considered to investigate processes from a broader perspective.^80^^,^^81^ Accordingly, the patterns described here should be interpreted as holobiont-level signals, with partner-specific origins yet to be resolved. This limitation is particularly relevant when interpreting interspecific differences, as the two coral species host distinct Symbiodiniaceae genera, which may differentially contribute to the observed metabolic profiles and stress responses.

The metabolic profiles of the two species under control conditions differed significantly, as shown in Figure 2, indicating substantial phenotypic variation in metabolic energy production pathways. Notably, *S. pistillata* exhibited higher relative abundances of metabolites associated with glycolysis and the TCA cycle, whereas *P. damicornis* showed higher relative abundances of metabolites linked to the pentose phosphate and purine metabolism pathways. Corals acquire most of their metabolic requirements from algal symbionts, which can supply most of a coral’s carbon needs through photosynthesis.^83^^,^^84^ However, little is known about the metabolic pathways that produce energy used by different coral species or in different compartments of the same organism. It has been observed that *Montipora capitata* polyps rely more on the glyoxylate cycle to derive carbon from internal storage compounds, such as lipids in their outer layer, while compartmentalizing the shunting of glucose to the PPP in their inner core.^85^ Moreover, it has been demonstrated that coral hosts’ capacity to rely on multiple metabolic energy sources, such as heterotrophic feeding, can influence their survival and recovery from stress.^86^ Indeed, different species employed alternative strategies to obtain metabolic energy, such as consuming biomass and lipids or increasing heterotrophic feeding of polyps, and this may also be reflected in interspecific differences in the electively adopted metabolic pathways for energy production.^87^ Furthermore, when corals experience thermal stress, their symbiotic algae may upregulate the production of protective compounds like flavonoids and lignans.^88^ These metabolites act as antioxidants and antimicrobial agents in order to improve coral resilience.^89^ Since differences in Symbiodiniaceae composition can influence coral susceptibility to bleaching and recovery, the metabolic pathways identified in the two species investigated in this study under control conditions may provide clues to differences in susceptibility to heat stress and may influence the strategies adopted to cope with bleaching conditions.^86^^,^^90^

The PCA analysis of the metabolites from both species, shown in Figure 3, revealed clustering of samples exposed to 1- and 3-day heat stress with control samples. Conversely, the samples exposed to 10 days of heat stress formed a distinct cluster, indicating divergent metabolic behavior compared with the other conditions. Indeed, consistent with its metabolic profile, *P. damicornis* did not show a significant change in bleaching status during the first 3 days of heat stress. On the other hand, *S. pistillata* exhibited significant bleaching after only 1 day of stress, despite no significant change in its metabolic behavior. It has been extensively demonstrated that the stress response in corals is a rapid process that involves multiple cellular compartments and depends heavily on the type of stress, the species under investigation, and the coral’s ecological history.^48^^,^^91^^,^^92^^,^^93^ Indeed, several studies have detected a gene response to stress even a few hours after the onset of stress conditions, providing evidence that corals quickly react to changes in their surrounding environment.^94^ Nonetheless, while gene expression data provide only putative insights into a system’s physiological response, metabolomics data reflect the system’s ultimate physiological response.^95^ Our results demonstrate that cellular responses at the biochemical and metabolic levels may be delayed relative to the onset of bleaching, as observed in *S. pistillata*, and that different species may exhibit distinct phenotypic patterns and response timing, thereby influencing the holobiont’s ability to recover.

Metabolic profiling of both coral species after 10 days of thermal stress, as shown in Figures 4 and 5, revealed a significantly different overall metabolic response compared with corals under control conditions and after 1 and 3 days of stress. In *P. damicornis*, together with significant bleaching, an overall increase in amino acid metabolism was observed. This complex metabolic process involves a series of biochemical pathways, with protein biosynthesis being a key outcome in scleractinian corals.^96^^,^^97^ Protein overexpression has been largely documented in corals under heat stress conditions, and a large number of protein biomarkers have been identified as critical contributors to corals’ stress response.^53^^,^^98^^,^^99^^,^^100^^,^^101^^,^^102^^,^^103^ For example, heat shock proteins (Hsps) play a crucial role in preventing and reducing the aggregation of other proteins damaged by heat or other environmental stress, and in assisting in the refolding or degradation of stress-damaged proteins.^104^ The overexpression of Hsps in heat stress conditions has been widely reported in several coral species, and it has been hypothesized that these proteins may act as a cellular defense against the effects of heat stress that may lead to bleaching, such as overproduction of ROS and related oxidative stress, and nutrient cycling impairment between host and symbionts.^21^^,^^28^^,^^36^^,^^105^^,^^106^^,^^107^ Other well-known stress responses involve several protein pathways, such as antioxidant enzymes, ubiquitin, and B-crystallin, which stabilize biological systems and eliminate ROS.^51^^,^^98^^,^^108^^,^^109^^,^^110^^,^^111^^,^^112^^,^^113^ The observed changes in free amino acid pools may also reflect altered host energy balance or shifts in host–symbiont nutrient exchange under thermal stress, rather than protein degradation per se, consistent with recent research describing early host starvation responses preceding bleaching.^114^^,^^115^ Moreover, previous studies have reported increased amino acid levels in the model anemone species *Exaiptasia diaphana*^116^ and in the hard coral species *Acropora aspera*.^86^ The authors suggested that, in addition to synthesizing new stress-response proteins, a higher concentration of amino acids in the cellular environment may be associated with protein autophagy. These amino acids can also be utilized to generate energy through the TCA cycle and oxidative phosphorylation (e.g., oxaloacetate and fumarate) and to produce sugar phosphate via gluconeogenesis.^86^^,^^116^ However, further investigation is needed to clarify the role of amino acid metabolism in corals under stress. Moreover, the significant increase in glutathione levels observed in samples stressed for 10 days relative to controls is consistent with an upregulation of the redox-buffering capacity in *P. damicornis*. Glutathione emerged from the untargeted metabolomics dataset as a redox-related metabolite significantly altered by thermal stress. Glutathione plays a key role in scavenging ROS and protecting proteins, lipids, and nucleic acids from oxidative damage. Similar increases in glutathione and related intermediates have been reported in thermally tolerant cnidarian holobionts, in which both host and symbiont contribute to redox homeostasis. In this context, the pattern observed here suggests a coordinated antioxidant adjustment within the holobiont, although the relative contributions of host and symbiont pools remain unresolved.^61^^,^^117^ Indeed, glutathione is a tripeptide involved in the detoxification of ROS, which are oxidized preferentially over biological molecules, thereby protecting the cellular environment from oxidative stress.^114^^,^^118^ Whether the increased level of glutathione should be attributed to the host or to the symbiotic components of the holobiont, or both, will need to be defined with future analyses, as it has been hypothesized that heat-resistant strains of *Hexaiptasia diaphana* may be able to obtain glutathione precursors directly from their algal symbionts.^116^ The increase in protein and peptide complex synthesis was also supported by increased one-carbon metabolism, as evidenced by high levels of adenosine, serine, methionine, S-adenosylhomocysteine, and 5′-methylthioadenosine, and by increased rates of ammonia recycling, as evidenced by high levels of urocanate, asparagine, histidine, serine, and glutamine. In addition, heat-stress-induced increases in energetic demand can be compensated for by amino acid degradation, and these enhanced antioxidant and catabolic reactions may reflect early metabolic stress responses associated with thermal tolerance rather than definitive markers of resilience.^114^ The metabolic profiling of *P. damicornis* and *S. pistillata* revealed an increase in amino sugar metabolism (hexosamine pathway, HBP), indicated by the high levels of N-Acetyl-D-glucosamine, uridine 5′-diphosphate (UDP-GlcNAc), and glucosamine 6-phosphate. The HBP is essential for the glycosylation of proteins and lipids. Therefore, these changes in the HBP pathway induced by the metabolic symbiotic relationship might be a strategy for both coral species to reduce heat stress and sustain survival; further analyses should investigate this. Taken together, the increase in amino acid metabolism and glutathione levels in *P. damicornis* is consistent with the oxidative stress hypothesis of coral bleaching, as these changes reflect enhanced antioxidant capacity and cellular stress responses aimed at mitigating ROS.^15^ At the same time, shifts in nitrogen-related pathways and amino acid turnover may indicate alterations in host-symbiont nutrient exchange, potentially linked to early stages of metabolic uncoupling.^114^ These combined responses suggest that the observed metabolic reprogramming may contribute to maintaining cellular homeostasis under thermal stress, even in the presence of symbiont loss. In contrast to *P. damicornis*, after 10 days of heat stress, glycolysis and the TCA cycle were reduced in *S. pistillata,* while, in turn, the increased levels of fatty acid metabolism could ensure energy reservoir maintenance. Given that corals primarily rely on these pathways to meet their metabolic demands under control conditions, with symbiont density restored in their tissues, we expected the original metabolic pathways to be restored as well. Indeed, *S. pistillata* showed a recovery of algal symbionts’ parameters after 10 days of stress, with symbiont cells’ density and Chl *a* concentration similar to control conditions. Our results indicate that such a recovery at the metabolic level did not take place, which is conceptually consistent with models proposing that altered nutrient exchange between host and symbionts under stress may favor symbiont maintenance or proliferation, as previously described in bleaching conditions.^15^^,^^17^^,^^25^^,^^26^^,^^114^ However, our data do not directly assess nutrient fluxes, so further analyses are needed to validate this hypothesis. This apparent decoupling between physiological recovery (i.e., restoration of symbiont density and chlorophyll content) and metabolic state suggests that the holobiont may remain in a post-stress metabolic configuration even after partial symbiont re-establishment. This condition could reflect a transitional state, in which newly established or recovering symbionts do not yet fully restore pre-stress nutrient exchange dynamics, or alternatively a shift toward a different metabolic equilibrium under stress. Such a pattern is consistent with scenarios of partial bleaching recovery or altered host-symbiont interactions, rather than a complete return to pre-stress homeostasis.^12^^,^^17^^,^^84^^,^^114^ In this context, *S. pistillata* seemed to exploit alternative metabolic pathways to sustain energy production under conditions of limited photosynthate availability, as previously hypothesized.^27^^,^^119^

Interestingly, compared to control conditions, high levels of palmitoleic acid were detected in *S. pistillata* after 10 days of thermal stress. Previous studies have reported higher levels of palmitoleic acid in corals adapted to extreme environments, such as the shallow waters of mangrove forests, compared to their reef counterparts, and these differences were linked to differences in Symbiodiniaceae profiles.^61^ While the specific role of these fatty acids in corals remains unclear, higher levels have been reported in corals inhabiting extreme or thermally variable environments and are associated with distinct *Symbiodiniaceae* assemblages. This monounsaturated fatty acid appears to be a prominent component of cnidarian lipid profiles, and its increase in *S. pistillata*, as further investigated, might reflect coordinated adjustments in both host and symbiont metabolism (Figure 5E).^64^ The reduction in glycolysis and TCA cycle intermediates, together with the reliance on alternative metabolic pathways, is consistent with the carbon limitation hypothesis, suggesting reduced availability of symbiont-derived photosynthates under stress conditions.^15^ Additionally, the observed increase in metabolites associated with the HBP in both coral species may be consistent with a common strategy to enhance protein and lipid glycosylation and to sustain cellular protection under thermal stress. However, the functional necessity of this pathway in modulating bleaching phenotypes requires experimental validation through targeted quantification and functional perturbation studies. In contrast, *P. damicornis* exhibited increased relative abundances of several TCA cycle intermediates under thermal stress, indicating a modulation of central carbon metabolism following symbiont loss.^119^ It is important to mention that *S. pistillata* and *P. damicornis* belong to the same family, Pocilloporidae, and despite being closely related from a genetic point of view, they showed an overall significantly different metabolic response to stress, strengthening the hypothesis that bleaching response is an individual-specific, rather than a species-specific process.^78^ It is important to mention that given that pathway interpretations, such as glycolisis and TCA cycle, are derived from a subset of metabolites detected within each pathway; these changes should be interpreted as indicative trends rather than definitive evidence of global pathway regulation.

In conclusion, this study investigated the metabolic profile of two coral species during 10 days of heat stress. The two species exhibited substantially different bleaching patterns during this period, with *P. damicornis* showing a marked decline in symbiont density and Chl *a* concentration, whereas *S. pistillata* showed an apparent recovery by the end of the 10-day period. Under control conditions, *S. pistillata* exhibited higher relative levels of glycolysis and TCA-cycle intermediates, whereas *P. damicornis* showed higher abundances in pentose-phosphate and purine-related metabolites (Figure 2B), which should be interpreted as baseline associations rather than definitive species-level metabolic strategies or indicators of pathway reliance. Both species showed a significant metabolomic response after 10 days of stress onset. In general, *P. damicornis* enhanced pathways dedicated to cellular protection, increasing its amino acid metabolism. By contrast, *S. pistillata* exhibited a different response, attempting to maintain alternative metabolic pathways. Despite having recovered from bleaching, *S. pistillata* altered its metabolic resources after 10 days of stress, suggesting that symbionts may contribute less to the coral’s metabolic demand under stress than under control conditions. Overall, metabolomics proved an efficient tool for investigating the strategies adopted by different coral species in response to heat stress, and it has the potential to provide insights into the diverse metabolic strategies corals may use to mitigate stress. Additionally, this study raises new questions about the role of coral host and symbiont metabolism in thermal tolerance, including how different symbiont clades can influence metabolic pathways across coral species and to what extent these pathways contribute to enhanced thermal tolerance. To answer these questions, future studies can leverage this innovative technique, which has been underutilized in coral research. Although the present metabolomic analysis was conducted at the holobiont level and thus cannot disentangle host from symbiont-derived metabolites, our results are consistent with the view that both partners contribute to the observed metabolic adjustments under heat stress. Previous multi-omics and metabolomic studies have shown that coral hosts and their Symbiodiniaceae can display distinct yet complementary responses to thermal stress, including differential regulation of antioxidant, central carbon, and lipid metabolism in heat-tolerant versus heat-sensitive partnerships. In some cases, thermally tolerant holobionts exhibit stronger host-side modulation of stress-response pathways, whereas sensitive combinations show more pronounced symbiont metabolic disruption. The increase in glutathione and amino acid metabolism observed here in *P. damicornis*, together with lipid remodeling and palmitoleic acid accumulation in *S. pistillata*, is therefore compatible with integrated adjustments of both host and symbiont compartments, even though their individual contributions remain unresolved. Future work combining tissue separation or cell-sorting approaches with compartment-specific metabolomics and transcriptomics will be necessary to attribute these signatures to specific partners and to determine how different host-symbiont combinations partition metabolic investment in redox balance, energy production, and membrane remodeling under heat stress.

### Limitations of the study

This study provides a detailed metabolomic characterization of thermal stress responses in two common Indo-Pacific corals, revealing clear and species-specific metabolic patterns that align with distinct bleaching trajectories. Because our approach was intentionally broad and exploratory, these findings should be considered a starting point, a framework that supports deeper mechanistic work rather than a final explanation of coral thermotolerance. From this perspective, our results provide robust descriptive evidence that informs focused, testable hypotheses for future investigation. Although the metabolites highlighted here, such as glutathione, palmitoleic acid, and hexosamine-related intermediates, were identified through an untargeted workflow, their integration with statistical analyses uncovered coherent biological signals. These patterns provide meaningful insights into pathway-level alterations associated with thermal stress, yet their confirmation through targeted quantification will be an important step toward validating the most promising candidate markers and refining their biological interpretation.

A further consideration concerns the biological scale of our measurements. The metabolomic profiles captured in this study reflect the combined response of the coral host, its Symbiodiniaceae symbionts, and further components of the symbiosis, such as the microbiome. Disentangling each partner’s contributions would undoubtedly yield greater mechanistic resolution. Nevertheless, the holobiont-wide patterns reported here remain ecologically relevant, as corals naturally face environmental stress as integrated biological systems rather than as isolated components. In this sense, the composite signatures we report reflect the true ecological reality of coral symbiosis. Our experiment was designed primarily to focus on metabolic dynamics and bleaching progression; therefore, other physiological indicators, such as long-term survival, calcification, oxidative stress biomarkers, or photosynthetic performance, were beyond its scope. Future studies that integrate metabolomics with these physiological measurements will be essential for determining whether the metabolic states identified here translate into measurable differences in coral resilience or susceptibility. Finally, several pathways highlighted in this work, including amino acid metabolism, redox buffering, hexosamine biosynthesis, and lipid remodeling, emerge as promising targets for deeper investigation. While our findings point to their potential involvement in thermotolerance, functional and manipulative experiments will be necessary to determine their causal roles and clarify how these metabolic processes contribute to the diverse strategies corals employ under thermal stress. Overall, this study establishes a coherent comparative metabolomic framework for two ecologically important coral species and identifies a suite of high-priority metabolic processes that warrant continued exploration. By articulating clear hypotheses and revealing key biochemical signatures associated with thermal stress, our work lays the foundation for future targeted, mechanistic research to understand and ultimately strengthen coral resilience in a warming ocean.

## Resource availability

### Lead contact

Requests for further information and resources should be directed to and will be fulfilled by the lead contact, Prof. Davide Seveso (davide.seveso@unimib.it).

### Materials availability

This study did not generate new, unique reagents, or materials.

### Data and code availability

Metabolomics raw data have been deposited at OSF Repository and are publicly available as of the date of publication at https://osf.io/t4xd9/overview?view_only=4276e8e53c294a5fa4165eae888ed2eb. Symbiodiniaceae ITS2 sequences are publicly available as of the date of publication at https://www.ncbi.nlm.nih.gov/nuccore/?term=PP264470-PP264485. Data reported in this paper will be shared by the lead contact upon request. This paper does not report original code. Any additional information required to reanalyze the data reported in this paper is available from the lead contact upon request.

## Acknowledgments

The authors wish to thank Dr. Valerio Isa and the staff of the Tropical Department of the Aquarium of Genoa (Italy) for their helpful technical and logistical support. Furthermore, the authors sincerely thank Dr. Roberto Giacchini for his essential help in his laboratory at the University of Milano-Bicocca. The authors thank the National Recovery and Resilience Plan (NRRP) Mission 4 Component 2 Investment 1.4—call for tender no. 3138 of December 16, 2021, rectified by decree n.3175 of December 18, 2021 of Italian Ministry of University and Research funded by the European Union – NextGenerationEU; award number, project code CN_00000033, concession decree no. 1034 of June 17, 2022 adopted by the Italian Ministry of University and Research, CUP H43C22000530001, project title “National Biodiversity Future Center—NBFC,” P.G.P. thanks the financial support through the Ramón y Cajal program (reference, RYC2023-044123-I) by the Spanish Ministry of Science, Innovation and Universities, the National Research Agency (MCIU/AEI/10.13039/501100011033), and the European Social Fund Plus (FSE+).

## Author contributions

Conceptualization, formal analysis, writing – original draft, and investigation, E.M.; Conceptualization, formal analysis, writing – original draft, and investigation, T.A.; methodology, formal analysis, data curation, and software, M.B.; methodology and formal analysis, Y.D.L.; methodology and formal analysis, E.B.; data curation, software, formal analysis, L.Z.; data curation, software and formal analysis, P.G.P.; supervision and project administration, D.P.; data curation, software and formal analysis, L.L.; resources, supervision, and methodology, S.L.; conceptualization, formal analysis, writing – review and editing, supervision, funding acquisition, validation, D.S.; supervision, funding acquisition, and resources, P.G.; supervision, resources, funding acquisition, writing – review and editing, investigation, and validation, D.G.

## Declaration of interests

The authors declare no competing interests.

## STAR★Methods

### Key resources table

**Table:** 

REAGENT or RESOURCE	SOURCE	IDENTIFIER
**Chemicals, peptides, and recombinant proteins**		

Water LC-MS CHROMASOLV	Honeywell, Charlotte, NC, USA	39253-1L, CAS 7732-18- 518,70 5
Acetonitrile LC-MS CHROMASOLV	Honeywell, Charlotte, NC, USA	34967-2.5L, CAS 75-05-8
2-Propanol LC-MS CHROMASOLV	Honeywell, Charlotte, NC, USA	34965-2.5L, CAS 67-63-0
Methanol LC-MS CHROMASOLV	Honeywell, Charlotte, NC, USA	34966-2.5L, CAS 67-56-1
*tert*-Butyl methyl ether CHROMASOLV	Honeywell, Charlotte, NC, USA	34875-2.5L, CAS 1634-04-4
Formic acid	Honeywell, Charlotte, NC, USA	CL00.1388.0050, CAS 64-18-6

**Critical commercial assays**		

DNeasy Blood and Tissue Kit (Qiagen, Hilden, Germany)	Qiagen, Hilden, Germany	DNA Extraction
Glutathione Assay Kit	Biovision, Inc., Milpitas CA, USA	K264-100

**Deposited data**		

Symbiodiniaceae (Dynoflagellates) ITS2 region DNA sequence	GenBank	PP264470-PP264485
Metabolomics raw data	OSF Repository	https://osf.io/t4xd9/?view_only=4276e8e53c294a5fa4165eae888ed2eb

**Experimental models: Organisms/strains**		

*Pocillopora damicornis*	Genova Aquarium	Aphia ID: 206953; World Register of Marine Species (https://www.marinespecies.org/aphia.php?p=taxdetails&id=206953)
*Stylophora pistillata*	Genova Aquarium	Aphia ID: 206982; World Register of Marine Species (https://www.marinespecies.org/aphia.php?p=taxdetails&id=206982)

**Software and algorithms**		

MassHunter ProFinder software (version 10.0.2)	Agilent Technologies, Santa Clara, CA, USA	
Mass Profiler Professional 15.1	Agilent Technologies, Santa Clara, CA, USA	
Agilent PCDL Manager (version B.08.00)	Agilent Technologies, Santa Clara, CA, USA	
MetaboAnalyst 5.0		
RStudio (2025.09.2 built 418)		Posit Softwares, PBC
IBM SPSS statistics ver. 29		IBM
MS-DIAL v. 4.90		
SIMCA 17 (Umetrics®, Umeå, Sweden)		

### Experimental model and study participant details

At the Aquarium of Genoa (Italy), a total of 80 coral nubbins (approximately 10 cm in length each, ±2 cm SD) of *Pocillopora damicornis* and *Stylophora pistillata* (40 fragments each) were obtained from eight large mother colonies (Figure S1A) in June 2024. These organisms originated in the northern Red Sea but have been bred in aquarium facilities at a constant temperature of 25 °C for more than 5 years. The nubbins were promptly fixed to supports using epoxy resin and transferred for acclimatization under controlled conditions in a 400 L tank (water turnover rate 120 L h^-1^) for 7 days. The tank was supplied with filtered seawater pumped from 50 m depth. Colonies were fed daily with *Artemia salina*. The temperature was set to 25 °C using a single heater (NEWA Therm, 300 W) and regulated by the aquarium’s automated temperature-control system. Corals were illuminated by two 96 W metal halide lamps (Sylvania, Domilux) at an irradiance of 170 ± 10 μmol photons m^−2^ s^−1^ (photoperiod was 10 h:14 h, light: dark). Further details of the experimental setup are provided in the supplementary materials (Section S1 and Figure S1). An additional 20 coral nubbins for each species, obtained from the same mother colonies and reared under the same conditions described above, were acclimatized in the same 400 L tank and then transferred into two 70 L Control tanks, in which the temperature was maintained at 25 °C for the whole duration of the experiment.

In order to carry out the treatments in duplicate, all fragments were evenly distributed, separating the two species into four 70 L treatment tanks (2 tanks for *P. damicornis* and 2 tanks for *S. pistillata*) containing water from the acclimatization tank, for a further acclimatization period of 14 days under the same conditions described above (Figure S1B). Water temperature in all experimental tanks was regulated by the Aquarium of Genoa’s automated life-support system, which continuously monitors and controls the temperature, maintaining it within ±0.5 °C of the target value. In addition to automated regulation, water temperature in each tank was manually verified twice daily using calibrated digital thermometers to ensure consistency among tanks and across the experimental period. Throughout the experiment, control tanks were maintained at 25 °C and heated tanks at 31 °C, with temperature fluctuations remaining within the defined ±0.5 °C tolerance. Daily checks of all other chemical and physical parameters were performed throughout the experiment, including pH, ammonium, nitrite, nitrate, and salinity (Table S1).

After the recovery period, 5 coral nubbins per species were collected from each tank before the onset of stress and considered control samples (Control). The seawater temperature was then increased from 25°C to 31°C (1 °C per day, Ramping phase) and maintained constant at 31 °C for 10 days (Stress phase) (Figure 1A). After reaching the maximum temperature, nubbins were collected at three different sampling times, particularly after 1 day, 3 days, and 10 days following the thermal stress (*n* = 5 per species, tank and sampling time) (Figure 1A).

One fragment (2 cm each) for each sampled nubbin was stored at −20 °C for chlorophyll quantification and Symbiodiniaceae density analysis. The remaining portions of each coral nubbin (approximately 8 cm) were first snap-frozen in liquid nitrogen and then promptly transferred to the University of Milano-Bicocca laboratories, where they were stored at −80 °C for metabolomics analysis.

### Method details

#### Chlorophyll *a* quantification and Symbiodiniaceae density

Coral tissue was blasted off from frozen coral fragments by directing airflow from a sterile 1,000 μL pipette tip connected via a rubber hose to an air compressor equipped with an air filter.^120^ Half of this tissue amount was used for chlorophyll *a* quantification. To do this, 5 mL of ice-cold phosphate-buffered saline was added, and the tissue slurry was centrifuged at 3600×*g* for 4 min and then homogenized with a syringe and needle. The remaining half of each tissue sample was stored at −20 °C and fixed in 4% formalin for Symbiodiniaceae counts.^121^ The supernatant was removed, and the remaining pellet was incubated in 100% acetone for 24 h at 4 °C in the dark. Following extraction, the sample was re-centrifuged at 3600×*g* for 4 min. The supernatant was used to determine chlorophyll *a* concentration from fluorescence measurements at 630, 663, and 750 nm, which were then applied to dinoflagellate-specific equations and normalized to coral surface area.^122^ The remaining skeletons of the coral fragments were soaked in 10% bleach and left to dry (48 h). The surface area of the fragments was measured using the paraffin wax-dipping method.^123^ The change in weight due to wax addition was compared against a standard curve of dipped clay cylinders of known surface area to calculate the skeletal surface area of each fragment.

The remaining tissue obtained by air blasting was fixed in 4% formalin and used to count Symbiodiniaceae cells from six independent hemocytometer counts (Improved Neubauer) under an optical microscope (Leica, France). Cell density was calculated from the surface area of the respective fragments.^124^

#### Symbiodiniaceae identification

The identity of the predominant Symbiodiniaceae genera in each mother colony was determined prior to the experiment to investigate the possible role of different symbionts in the stress response of the holobiont. Specifically, DNA was extracted from eight *P. damicornis* and eight *S. pistillata* colonies with the DNeasy Blood and Tissue Kit (Qiagen, Hilden, Germany), and a portion of the ITS2 region was amplified using the primers SYM_VAR_5.8S2 and SYM_VAR_REV, as described in Hume et al.^125^^,^^126^ PCR products were checked by electrophoresis on 1.5% agarose gels, purified with Illustra ExoStar (GE Healthcare, Chicago, IL, USA), and sequenced using an ABI 3730xl DNA Analyser (Applied Biosystems, Carlsbad, CA, USA). The resulting chromatograms were checked, corrected, and assembled using Geneious 7.1.9 (Biomatters, Auckland, New Zealand), and the consensus sequences were deposited in GenBank with the accession numbers PP264470-PP264485. Finally, the Basic Local Alignment Search Tool (BLAST) was used to compare the obtained sequences with the NCBI database.

#### Metabolite extraction

Corals were rinsed with 0.9% NaCl to remove residual frozen seawater, then dried on filter paper. We ensured that this preliminary process took less than 1 min to prevent the coral tissues from thawing. These were then brushed off from frozen coral fragments using airflow (as described in the previous paragraph) into a ceramic mortar cooled on dry ice.^120^ Coral tissues were collected from the mortar and placed in a pre-weighed tube to determine their weight. 100 μL of ice-cold 50:50 methanol-water was added to 10 mg of coral tissue, and the samples were homogenized with a bead mill at 30 Hz for 4 min (TissueLyser II, Qiagen, Hilden, Germany). After a 10-min incubation at −80 °C, 320 μL of ice-cold methanol was added to 100 μL of homogenate, and the samples were vortexed for 2 min. Subsequently, 80 μL of methyl-*tert*-butyl ether (MTBE) was added, and the samples were shaken at 4 °C for 1 h. The samples were then centrifuged at 12,000*g* for 10 min 400 μL of supernatant was recovered in a flat-bottom glass insert and evaporated for 2:45-3 h in a centrifugal vacuum concentrator (Concentrator plus/Vacufuge plus, Eppendorf, Hamburg, Germany).

#### LC-MS metabolic profiling

Dried samples were resuspended in 150 μL of LC-MS-grade water and then analyzed by UHPLC-QTOF mass spectrometry. For each experimental condition, five independent biological replicates were prepared, and each biological replicate was measured in triplicate technical injections. LC separation was performed using an Agilent 1290 Infinity UHPLC system and an InfintyLab Poroshell 120 PFP column (2.1 × 100 mm, 2.7 μm; Agilent Technologies, Santa Clara, CA, USA). Mobile phase A was water with 0.1% formic acid. Mobile phase B was acetonitrile with 0.1% formic acid. The injection volume was 10 μL, and LC gradient conditions were: 0 min: 100% A; 2 min: 100% A; 4 min: 99% A; 10 min: 98% A; 11 min: 70% A; 15 min: 70% A; 16 min: 100% A with 2 min of post-run. The flow rate was 0.2 mL/min, and the column temperature was 35 °C. MS detection was performed using an Agilent 6550 iFunnel Q-TOF mass spectrometer with a Dual JetStream source, operating in negative-ion mode. MS parameters were gas temp: 285 °C; gas flow: 14 L/min; nebulizer pressure: 45 psig; sheath gas temp: 330 °C; sheath gas flow: 12 L/min; VCap: 3700 V; Fragmentor: 175 V; Skimmer: 65 V; Octopole RF: 750 V. Data were acquired from m/z 60 to m/z 1050. An active reference mass correction was performed using a second nebulizer, with masses at m/z 112.9855 and 1033.9881, dissolved in mobile phase 2-propanol:acetonitrile:water (70:20:10 v/v). Along with the sample, 2 μL of 4-nitrobenzoic acid was injected as an internal standard.

#### Glutathione quantification

Glutathione levels were measured using the Glutathione Assay Kit (Biovision, Inc., Milpitas, CA, USA). Briefly, 100 μL of Glutathione Assay Buffer was added to 40 mg of coral tissue. As mentioned above, the samples were homogenized with TissueLyser and processed according to the manufacturer’s protocol. Glutathione was detected by fluorescence at excitation/emission wavelengths of 340 nm/450 nm, respectively, using a Cary Eclipse Fluorescence Spectrophotometer (Agilent Technologies).

### Quantification and statistical analysis

#### Metabolomics data analysis

Data analysis was performed with MassHunter ProFinder software (version 10.0.2) (Agilent Technologies). Data preprocessing was performed using the Batch Targeted Feature Extraction tool and Agile 2 algorithm. This software assigned identities to metabolites by searching an in-house compound database built with Agilent PCDL Manager (version B.08.00) using the metabolite formula and its corresponding retention time, with a score >75. The library was developed by integrating authentic chemical standards to ensure accurate retention time assignment and confident annotation. Only metabolites with reliable identification based on retention time matching the library annotation were included in downstream analysis. Statistical analysis was performed using Mass Profiler Professional 15.1 (Agilent Technologies). Metabolite annotation was performed within an untargeted LC-MS workflow. Feature annotation was based on accurate mass, isotopic pattern, and database matching against publicly available metabolite libraries. Accordingly, metabolite identities correspond to Metabolomics Standards Initiative (MSI) levels 2–3. Specialized metabolites (e.g., flavonoids, coumarins, lignans, stilbenes) were annotated at the compound-class level based on accurate mass features and database classification, with confidence further supported by chemical-class enrichment analyses performed using MetaboAnalyst 5.0. These compounds are therefore reported as putative members of their respective chemical families rather than as fully confirmed molecular structures. Internal standards were systematically included in all analytical sequences. Peak areas were normalized to the internal standard signal (4-nitro-benzoic acid std) using the normalization algorithm implemented in Mass Profiler Professional (MPP) prior to statistical analysis. Pooled quality control (QC) samples and blanks were included to monitor analytical stability and background signals throughout the LC-MS runs. Following internal standard normalization, data were Pareto-scaled before multivariate and univariate analyses. Metabolite abundances are reported as relative intensities rather than absolute concentrations. For each metabolic pathway presented in the Results, we report the number of metabolites detected and the number that show statistically significant differences between treatments. Raw data were transformed in *log2* scale and normalized using Pareto scaling. Data were then filtered, retaining only entities present in at least 80 percent of samples for a given condition. Statistical analysis was performed by applying an unpaired *t* test or One-way ANOVA analysis with a *p-*value cut-off of 0.05, followed by a multiple testing correction using the Benjamini-Hochberg FDR method. Principal component analyses were performed on conditions based on One-way ANOVA entity lists. Data visualization of significant entities was performed using a hierarchical clustering algorithm.

#### Spectral alignment and annotation of chemical entities by MS-DIAL

Samples were further analyzed using MS-DIAL v. 4.90 against two comprehensive databases, namely Fienh’s Vaniya Natural Products and the Global Natural Products Social Molecular Networking databases. The annotation of chemical features was achieved by setting the following parameters: MS1 tolerance = 0.05 Da, MS2 tolerance = 0.1 Da, MS1 m/z range = 100–1200 Da, MS2 m/z range = 100–1200 Da, total score alignment = 80%, peak count filter = 12.3%; only features being present in at least 80% of replicates were considered, and the blank-based features were removed.

#### Multivariate statistical analysis

Following annotation, multivariate statistical analysis was performed on the post-processed raw abundances, which were log2-transformed, adjusted to the 75th percentile, and baselined to the median of all samples. Unsupervised statistics, in terms of hierarchical cluster analysis (HCA; Euclidean distances, Ward’s linkage rule), and principal component analysis (PCA) were performed by Mass Profiler Professional (Agilent). Additionally, volcano analysis was performed using the same software to select Bonferroni-corrected features that met fold-change thresholds > |2| and statistical significance in paired t-tests (*p*-value threshold = 0.05), with thermal stress compared with control. The volcano-filtered features were then subjected to Chemical Similarity Enrichment Analysis for Metabolomics (ChemRICH),^127^ a statistical enrichment approach based on chemical similarity, providing the significantly altered chemical classes associated with heat stress. Supervised statistical modeling was performed using SIMCA 17 (Umetrics, Umeå, Sweden) via partial least squares discriminant analysis (PLS-DA). The quality of predicting models was assessed through two key parameters, i.e., goodness-of-fit, R^2^Y, and goodness-of-prediction, Q^2^Y (assuming >0.5 values for robust predictive models), and statistical validation was achieved through cross-validation analysis of variance (CV-ANOVA) and permutation test (*n* = 100). The obtained PLS models were further used to identify discriminant features, as indicated by variable importance in projection (VIP) markers, with a VIP score threshold of 1.0. Chemical overrepresentation analysis of VIP markers was performed using MetaboAnalyst 6.0, selecting the main-class metabolite set library. Boruta feature selection was performed using the R package Boruta (Wrapper Algorithm for All Relevant Feature Selection, v. 8.0.0),^128^ which considers 200 random forest models to reduce variability associated with non-informative factors and retains features directly involved in the effect of thermal stress.

## Supplemental information

Document S1. Figures S1–S6

Table S1. Aquarium water parameters details

Table S2. Specialized metabolic dataset

Table S3. VIP markers related to PLS-DA models

Table S4. Chemically enriched metabolites, deriving from ChemRICH analysis
