# Supplementary material for: Thermal stress-induced metabolic reprogramming in two hard coral species
Source: iScience. 2026 Jun 8;29(6):116207. doi: 10.1016/j.isci.2026.116207 (PMC13259630; doi:10.1016/j.isci.2026.116207)
Supplement: Document S1. Figures S1–S6 [file mmc1.pdf]

iScience, Volume *29*

Supplemental information

Thermal stress-induced metabolic reprogramming in two hard coral species

Enrico Montalbetti, Tecla Aramini, Marcella Bonanomi, Yohan Didier Louis, Elisa Brivio, Leilei Zhang, Pascual Garcia Perez, Danilo Porro, Luigi Lucini, Silvia Lavorano, Davide Seveso, Paolo Galli, and Daniela Gaglio

Supplementary Materials

S1 – Technical description of the Genoa Aquarium experimental set-up

The coral colonies sampled come from the tanks of the Genoa Aquarium, where the water system collects seawater from 200 m outside the Foranea dam of the port of Genoa at 50 m depth. The collected water is pumped through the filtration system made of 2 sand filters and one UV filter, used for disinfection. After the filtration, the seawater is stored inside 4 accumulation tanks (200 m^3 each). If the results of the analyses show that the chemical-physical parameters (Ph, salinity, ammonia, nitrites, nitrates, and phosphates) are optimal for the aquarium, the seawater of one accumulation tank is pumped into a mixing basin, where the water is kept in constant motion. After further UV filtration, the water is pumped from the mixing basin to all the aquarium tanks. During the day, from 8:00 to 16:00, the water is pumped from the mixing basin to the aquarium tanks with a flow of 1 L every 30 seconds, so the tanks are considered as a semi-open system (the tanks are considered as a closed system from 16:00 to 8:00). In the tanks, used for the sampling (3 x 1 x 0.7 m; 3100 L; composed by acrylic and glass resin) in which the experiment was carried out, the water is uptaken by a pump (Astralpool, Victoria Plus) with a 24-hour flow rate of 8 m^3/h (to ensure complete water change every about 30 minutes) and reinserted into the tank after passing through the filtration system. The filtration system is composed of a sand filter (Astralpool Artic, filtering particles from 0.4 to 2 mm) and a UV filter (Panaque 750 s AB 4 lamps of 40W). The water passage through the UV filter is instantaneous since water passes with a flow equal to 8 m^3 per hour. Two liters of water containing a solution of the algae *Tetraselmis* and zooplankton belonging to the Phylum *Rotifera* (the average concentration of zooplankton is 250 individuals/ml and the average dimension is 0.5 mm) are placed daily inside the tanks to feed the corals. Both Algae and zooplankton are farmed inside 80 L cylindrical tanks made of plexiglass. Furthermore, twice a week, 20 g of food mixture is placed daily in the tanks to feed the corals; this mixture is composed of 70% silverside fish (5 cm in length) and 30% carrots, while the next day, the mixture is composed by 70% of mussels and 30% of courgettes. To facilitate the calcification of the coral skeleton, 50 L of water containing 500 g of calcium hydroxide are also placed into the tanks.

Supplementary Figures

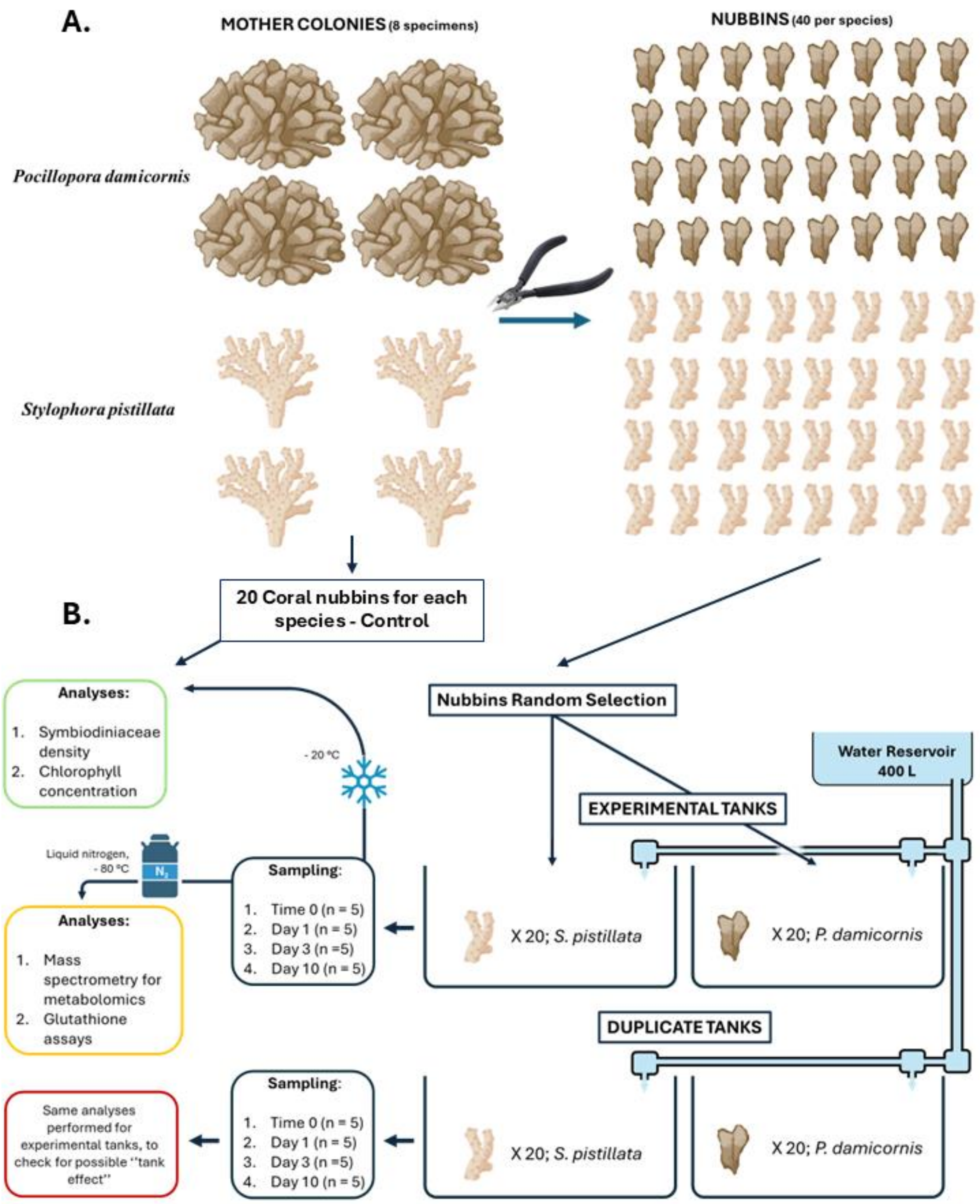

Figure S1. Schematic representation of the [A] experimental plan and [B] the analyses performed on *S. pistillata* and *P. damicornis* samples.

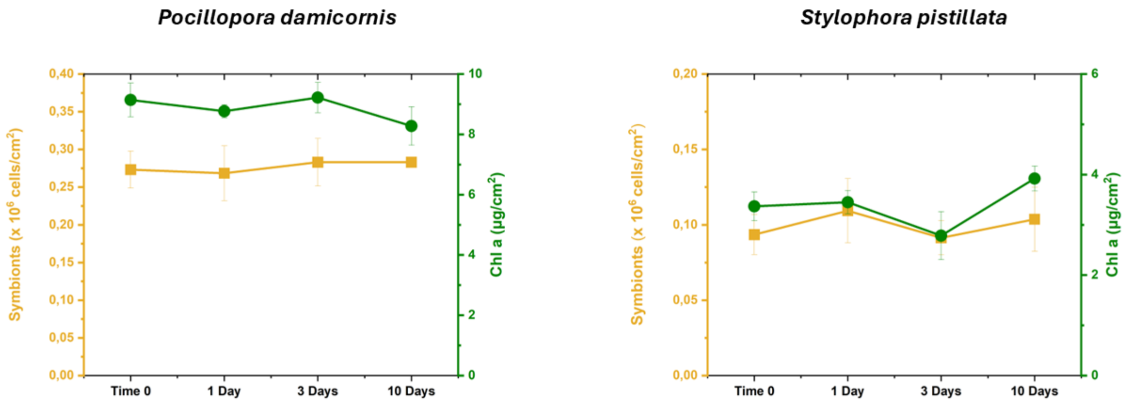

Fig. S2. Symbiodiniaceae density and chlorophyll *a* concentration in *P. damicornis* and *S. pistillata* control corals maintained at 25 °C and sampled at time 0, 1, 3, and 10 days. No significant temporal changes were detected, indicating that experimental duration alone did not affect bleaching-related parameters.

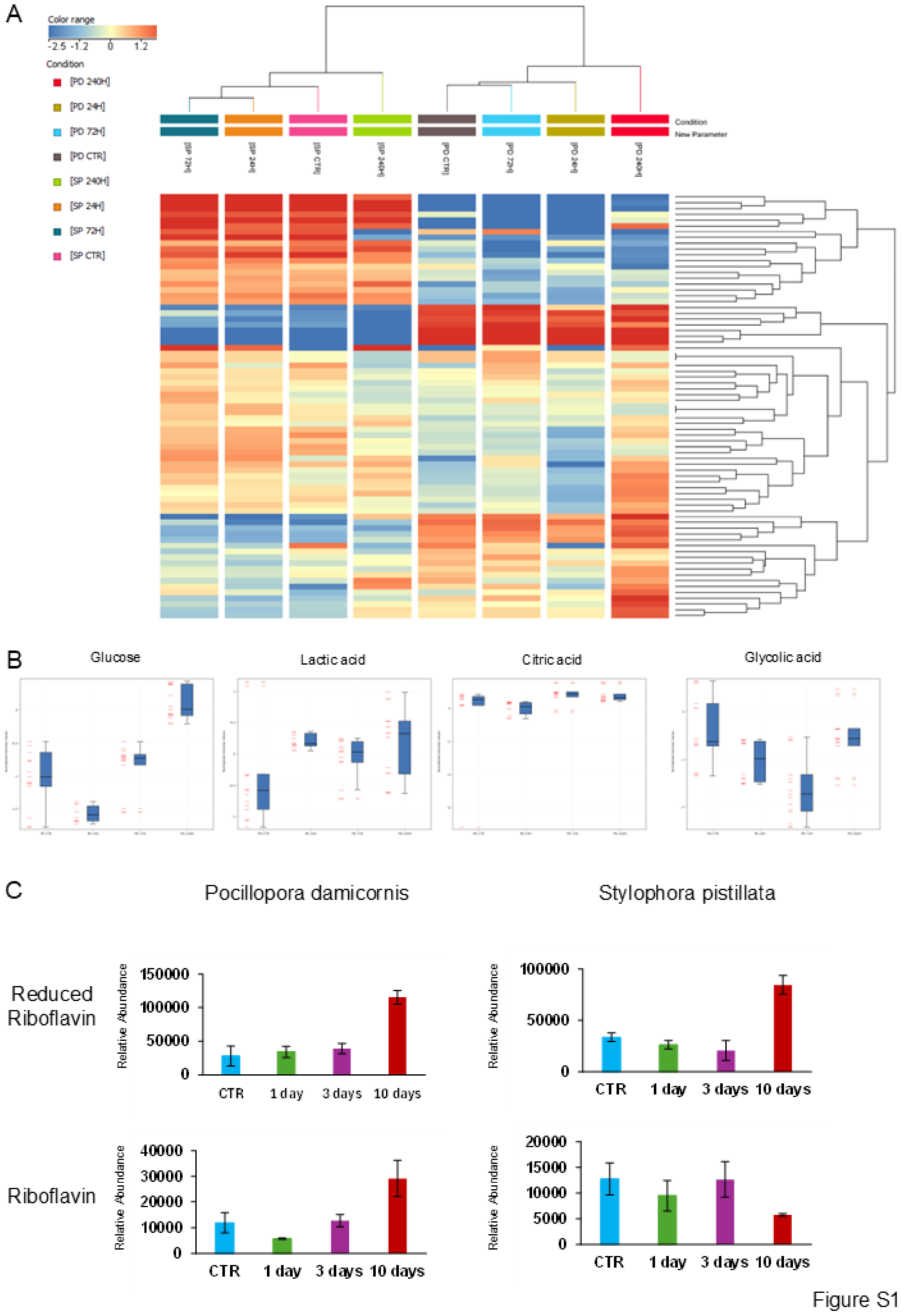

Figure S3. [A] Metabolomic profile of *S.pistillata* and *P.damicornis* at different time points. [B and C] Detail of specific metabolite expression in the two species at different time points. Details can be found in the Manuscript at paragraph 3.3.

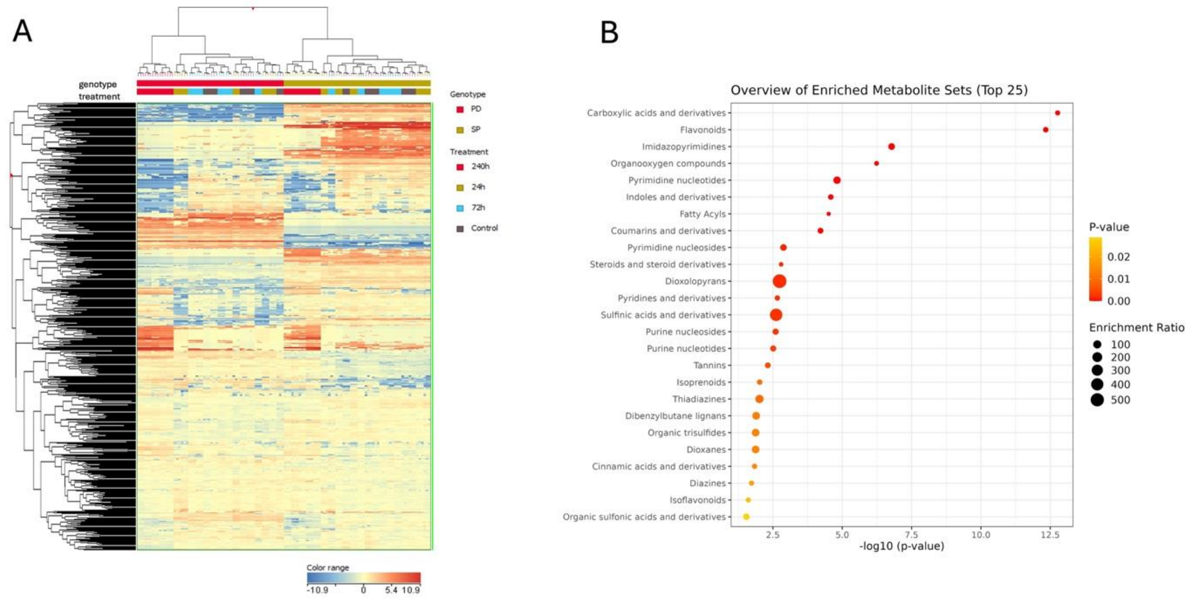

Figure S4. [A] The hierarchical cluster analysis of the two species of corals (PD: *Pocillopora damicornis* and SP: *Stylophora pistillata*). [B] The enrichment analysis reporting the 25 top enriched classes detected in the coral samples.

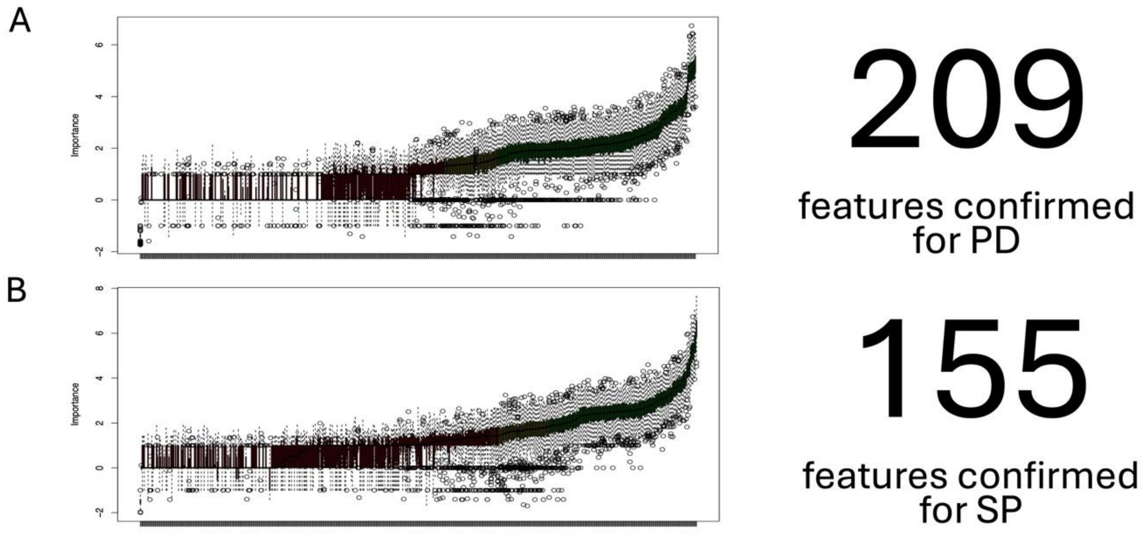

Figure S5. Boruta feature selection results for [A] *Pocillopora damicornis* (PD) and [B] *Stylophora pistillata* (SP) corals.

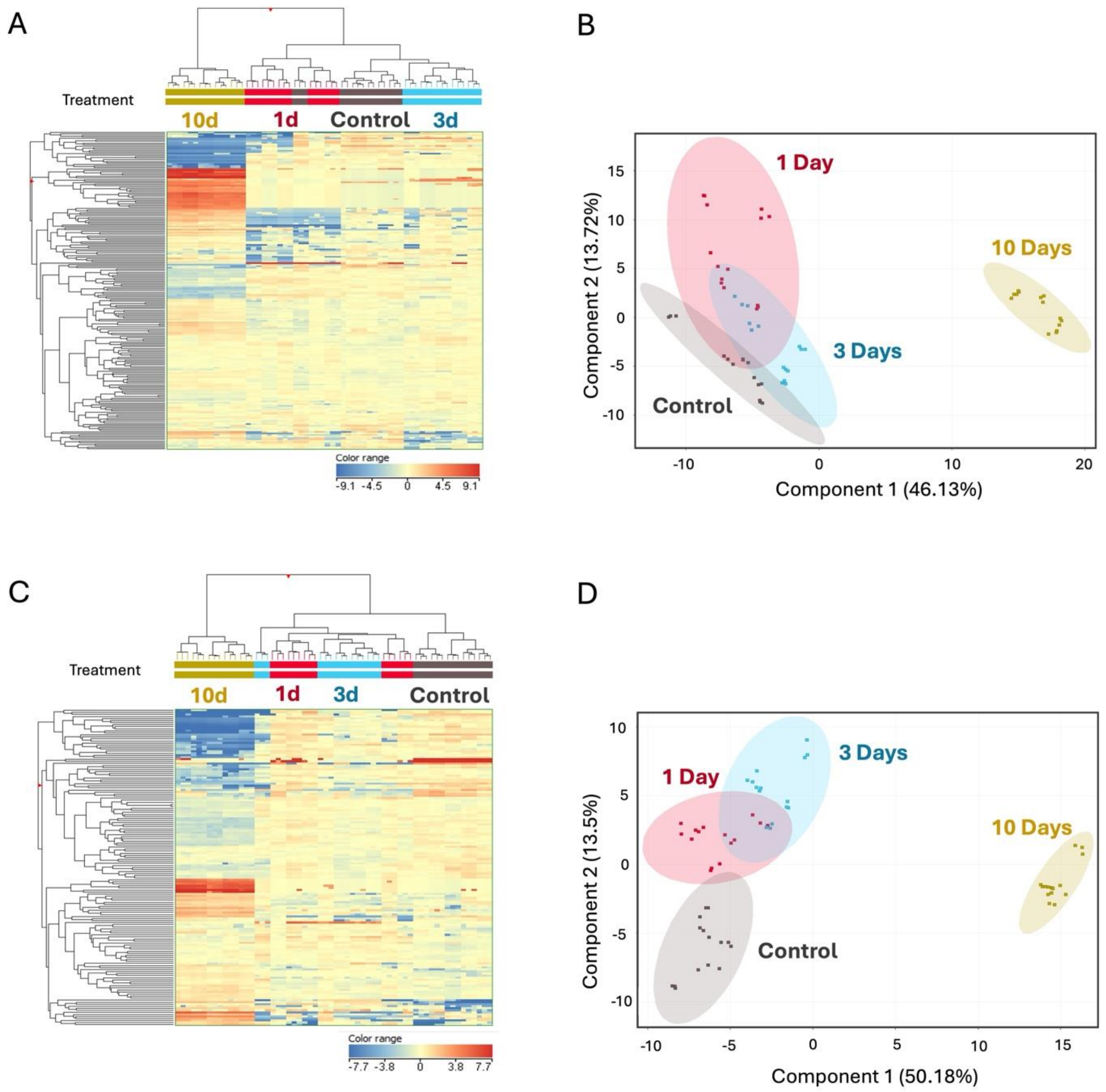

Figure S6. The hierarchical cluster analysis and principal component analysis of the two coral species. [A and B] for *Pocillopora damicornis* and [C and D] for *Stylophora pistillata*.
